# A Novel Lubricant Based on Covalent Functionalized Graphene Oxide Quantum Dots

**DOI:** 10.1038/s41598-018-24062-2

**Published:** 2018-04-11

**Authors:** Andreas Wolk, Marta Rosenthal, Stephan Neuhaus, Klaus Huber, Katharina Brassat, Jörg K. N. Lindner, Richard Grothe, Guido Grundmeier, Wolfgang Bremser, René Wilhelm

**Affiliations:** 1 University of Paderborn, Department of Chemistry, Technical Chemistry, Warburgerstr. 100, 33098 Paderborn, Germany; 2 University of Paderborn, Department of Chemistry, Organic Chemistry, Warburgerstr. 100, 33098 Paderborn, Germany; 3 University of Paderborn, Department of Chemistry, Physical Chemistry, Warburgerstr. 100, 33098 Paderborn, Germany; 4 University of Paderborn, Department of Physics, Warburgerstr. 100, 33098 Paderborn, Germany; 5 University of Paderborn, Department of Chemistry, Technical Chemistry, Warburgerstr. 100, 33098 Paderborn, Germany

## Abstract

Dodecyl amine edge functionalized few-layer graphene oxide quantum dots were synthesized in good yields. The covalent functionalization was demonstrated with NMR and AFM-IR. The resulting structure and particle size was measured with AFM and HRTEM. The thermal stability of the compound was investigated and showed a stability of up to 220 °C. The modified graphene oxide quantum dots showed excellent solubility in various organic solvents, including ethers, methanol, toluene, *n*-hexane, heptane, xylene, dichloromethane and toluene. The stability of a resulting toluene solution was also proven by static light scattering measurements over several days. The excellent solubility gives the possibility of an efficient and fast spray application of the functionalized graphene oxide quantum dots to steel surfaces. Hence, the macroscopic friction behavior was investigated with a Thwing-Albert FP-2250 friction tester. A thin film of the dodecyl amine functionalized graphene oxide quantum dots on steel lowered the friction coefficient from 0.17 to 0.11 and revealed a significant corrosion inhibition effect.

## Introduction

Controlling the friction performance of mechanical contacts like bearings and gears minimize the energy cost and increases the lifetime of everyday mechanical components^1^. Typically, lubricants are used to lower the frication of mechanical sliding contacts. Often the lubricant has also the task to prevent corrosion. It has been shown, that functionalized graphene and graphene oxide particles are able to inhibit corrosion^2–4^. Lubricants can be separated in liquid and solid lubricants. As liquid lubricants oils are commonly used while for solid lubricants graphite or molybdenum sulfite^5^ are applied. For many lubricants amines are the basic materials^6,7^. Recently, there have been reports that graphene and graphene oxide could be the base for solid and liquid lubricants^8–11^.

Graphene is a two dimensional, single-atom-thick sheet of hexagonally constructed sp^2^-bonded carbon atoms^12,13^. It has attracted much attention in recent years due to its exceptional properties such as thermal and mechanical stability as well as electrical and optical activity^14–19^. Graphene with an average lateral dimension of 3–30 nm has been applied as graphene quantum dots in photoluminescence experiments^20–24^. Larger graphene sheets of up to 100 nm have been also classified as graphene quantum dots in this respect^23^. In other applications graphene sheets below 100 nm are defined as graphene nanosheets while graphene sheets larger than 100 nm are termed graphene microsheets^12,25^. If 2 to 5 graphene layers are dominating, the material has the term few-layer graphene while with up to 10 layers the name multi-layer graphene is used to define the material^12^. Graphene quantum dots have been so far mainly considered for sensing applications^26^. In addition, carbon quantum dots have found application in sensing, nanomedicine, photocatalysis and electrocatalysis^27^.

A standard procedure to obtain graphene from graphite is to oxidize the latter to graphene oxide and transform it to reduced graphene oxide. However, reduced graphene oxide does not fully exhibit the behavior of graphene. Reduced graphene oxide sheets have defects in their sheet structure^28^. Furthermore, while graphene oxide often possess a C/O ratio of 4:1 reduced graphene oxide has often a ratio of 12:1^25^. A further alternative to obtain graphene sheets is to stabilize and increase their solubility via chemical functionalization. The functionalization of graphene, graphene oxide and reduced graphene oxide can influence the behavior of these materials^28^. Particularly the solubility in different solvents and hence their processability can be increased via functionalization^29,30^. Especially the surface functionalization can increase the solubility and also stabilize single graphene sheets. However, this introduces sp^3^ centers on the graphene sheets, which can influence the behavior of graphene^29,30^. In consequence, the graphene sheets incorporate oxygen containing groups (-OH, Epoxy, -COOH) which can be used to modify graphene oxide sheets with small organic molecules like octadecylamine (ODA)^31^, 5-(4-aminophenyl)-10,15,20-triphenyl porphyrin (TPP)^32^ or 2-(4-aminophenyl)ethanol^33^. The combination of amines with graphene oxide has shown a good performance in solid lubrification and additives for liquid lubrification^34,35^. The common technique for the assembly of the solid lubricants is a layer by layer assembly where the friction coefficient could change^36^. For example, a friction coefficient of 0.35 for a polymer vs. steel changed to 0.02 under nitrogen with a 300 nm thick polyethyleneimine/graphene oxide film. Under air a higher friction coefficient of 0.10 was observed^34^.

The behavior of graphene is also dependent on the size of the graphene sheets. If few- or multi-layer graphene is present in the material the solubility decreases. In addition, a decrease of the sheet size will aid to exfoliate graphene sheets. The most common approach to obtain graphene nanosheets from graphite is via electrochemical exfoliation^37^ and by a modified Hummers method^38^. In addition, a modified procedure was reported by Haino *et al*. where graphite was treated with H_2_SO_4_ and HNO_3_ at 120 °C for 24 h which resulted in edge carboxylated graphene quantum dots with a diameter of ca. 21 nm^39^. Functionalization of the graphene quantum dots with benzyl amine derivatives resulted in material soluble in polar solvents like ethyl acetate or dichloromethane^39^. Hence, a functionalization or disruption of the surface of the graphene sheets was prevented^39^. Alternatively, graphene quantum dots can be isolated from coal^40^. Furthermore, graphene quantum dots were prepared from CX-72 carbon black in high yields and were applied successfully as probes for cellular imaging and have high potential in optoelectronic devices^41^. The utilization of carboxylic acid functions present in carbon nanoparticles provides a straightforward route to covalent functionalization with amines via the formation of an amide^42^. The reaction is easy to perform and the resulting amide functions are more resistant to hydrolysis compared to ester functions, since the NHR moiety is even a poorer leaving group than OR^43,44^.

Graphene quantum dots are considered to be the next generation carbon based nanomaterial. This is due to their outstanding physical, chemical and biological properties. In the present study we emphasized that the small size of the graphene quantum dot particles will ensure a good adsorption on the surface, especially with defect and fraction present on the natural surface of steel. In addition, since functionalized graphene oxide quantum dots have not been applied as lubricants so far, we were interested in the functionalization of graphene oxide quantum dots and to influence their behavior as lubricants for steel. In this process, a high solubility of graphene material in different solvents would be beneficial. The ability to predominantly functionalize the edges of graphene sheets leaves the sheet structure intact and the less sp^3^ centers are present in the sheets the more the original electronic behavior of the graphene nanosheets is preserved.

## Experimental Details

### General Experimental

All reagents and solvents were purchased from commercial sources like *Fluka AG*, *Merck AG*, *Lancaster*, *Alfa Aesar*, *Riede*l *de Haën* and *Sigma Aldrich*. Unless specified otherwise the reagents were further purified by standard procedures. Graphite (flakes, +100 mesh particle size ($\ge$85%)) was purchased from *Sigma Aldrich*. Test panels Gardobond® cold-rolled steel were purchased from *Chemetal*. ^1^H NMR spectra were measured at room temperature by Avance 500 from *Bruker* using methanol as internal standard. Fourier transform infrared (FTIR) and attenuated total reflection (ATR) spectra were measured with VERTEX 700 by *Bruker*. Raman spectra were recorded by InVia spectroscope in combination with DM2500 M-microscope from *Leica Microsystems*. GQDs **5** were dissolved in toluene to be dropped on a mica-wafer or carbon-pallets for atomic force microscopy (AFM) by Veeco Dimension Icon from *Bruker* or for scanning electron microscopy (SEM). To prove the functional groups on the GQDs all products were scanned by thermo gravimetric analyzer-mass (TGA-MS) spectroscopy TGA/SDTA851 by *METTLER TOLEDO*. X-ray diffraction (XRD) pattern was measured at room temperature using AXS D505 by *Bruker*. Tribological studies of steel flat samples against coated flat steel samples were performed with a Thwing-Albert FP-2250 Friction/Peel tester with a 2 kg force load cell. The sled has a contact area of 6 cm × 6 cm and a weight of 500 g. The distance between the sled and the load cell is 10.2 cm. The sled was placed on a clean steel surface and moved with a speed of 50 cm/min. The friction coefficient was calculated automatically. Transmission electron microscopy (TEM) was performed at 200 kV with a probe-side Cs-corrected TEM (JEOL JEM-ARM200F), equipped with a cold field emission gun, a post-column energy filter (GIF Quantum ER, Gatan) and a JEOL energy dispersive X-ray spectroscopy (EDS) system. For this purpose, the toluene GOQs **5** suspensions (see below) were drop casted on a carbon coated Cu TEM grid. Even though it is known that at 200 kV graphene is destroyed by knock-on damage, we observed that the GQD were long enough stable in order to investigate them at this acceleration voltage. A similar observation was reported by Wilson and Sloan^45^.

### Preparation of graphene oxide quantum dots (GQD_COOH_) 2

Oxidized graphene quantum dots (GQD_COOH_) **2** were synthesized in the presence of 65% sulfuric acid and 95% nitric acid (3:1)^39^ from Graphite (flakes, +100 mesh particle size (≥85%)), purchased from Sigma Aldrich. The reaction mixture was treated with ultrasonic sound for 3 h to give a homogenous suspension. Afterwards the reaction was stirred at 120 °C for 1 d. The resulting product was neutralized with sodium hydroxide solution and washed with deionized water by use of ultrafiltration (membrane filter, 0.2 µm FG, FLUOROPORE^TM^) to give GQD_COOH_ **2**.

### Procedure to prepare GQD_COCl_ 3

Under inert gas conditions dry DMF (0.25 mL) was given slowly and dropwise to a reaction mixture of GQD_COOH_ **2** (5 g) and oxalyl chloride (63 mL, 0.74 mol, 19.5 eq.). To slurry the reagents the reaction was treated with ultrasonic sound under reflux for 3 h. Subsequently the suspension was stirred for 3 d under reflux. The residual oxalyl chloride was removed by redestillation to give GQD_COCl_ **3** (5.27 g) as grey powder, which was directly converted to the next step.

### Synthesis of 5

GQD_COCl_ **3** (0.5 g), NEt_3_ (3 mL), amine **4** (3.7 mmol, 1 eq.) and DMAP (4.5 mg, 0.037 mmol, 0.01 eq.) were added to dry DMF (10 mL) under nitrogen. The reaction mixture was stirred for 3 d under reflux at 110 °C under an N_2_-atmosphere. After completion of the reaction dichloromethane (5 mL) was added. The product was washed with acidulated (1 N HCl), bidestilled water (3 × 20 mL) and extracted with diethyl ether (3 × 30 mL) by using hydrophobic amine. Products functionalized with hydrophilic amine were purified by column chromatography using MeOH/CHCl_3_ as eluent. The degree of amide formation was determined by the content of nitrogen and oxygen by elementary analysis (w.%) (C = 71.9%; N = 6.5%; O = 11.3%; H = 11.4%), which corresponds to 80 mol%.

### Spray coating

Commercial Gardobond® cold-rolled steel sheets were cleaned by emerging in ethyl acetate. Before application, the steel sheets were dried for 1 h at room temperature. Thereafter, the spray deposition of the GQD **5** in toluene (0.1 g/L) was performed by using a commercial airbrush (Revall) supplied by N_2_ at 2.5 bar. The steel sheets were coated at a substrate-nozzle distance of 30 cm. Between two spray cycles the steel sheets were dried for 5 min at room temperature. The corrosion behavior was investigated using a steel sample with 20 spray cycles, which was emerged for 1 h in sea water (based on DIN 51358).

### Electrochemical impedance spectroscopy (EIS)

measurements were performed with a Reference 600 (Gamry, Germany) potentiostat. A gold wire and a Ag/AgCl electrode were used as counter and reference electrodes, respectively. Impedance data were collected in artificial seawater for a frequency of 0.1 Hz by superimposing a 10 mV AC voltage at open circuit potential for 1 h.

### Preparation of seawater

25 g sodium chloride, 11 g magnesium chloride*6 H_2_O, 4 g sodium sulfate and 1 g calcium chloride were dissolved in 1 L of water. The mixture was thoroughly stirred and adjusted by dropwise addition of 0.1 N sodium carbonate (Na_2_CO_3_) solution.

## Results and Discussion

**Figure 1:**
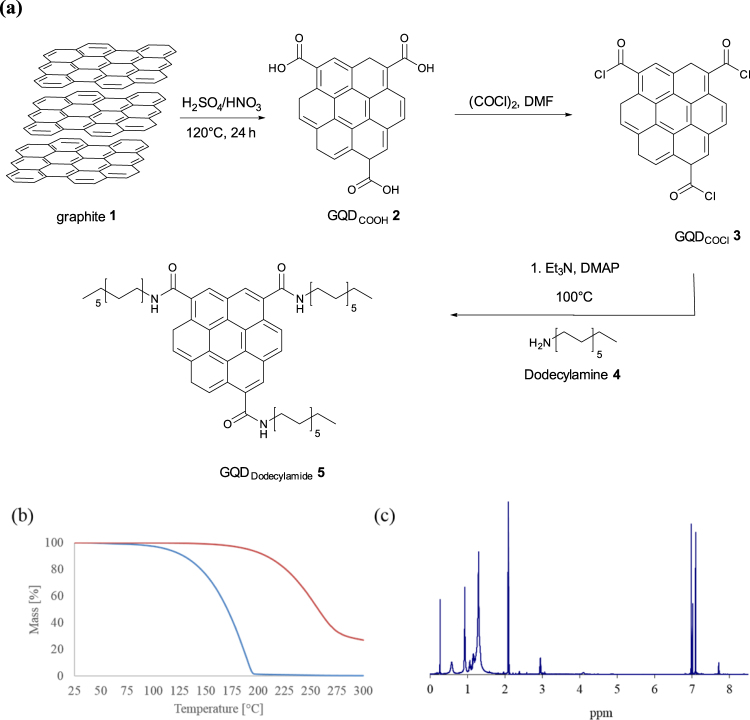
(**a**) Synthesis of covalent functionalized GQD with dodecyl amine, (**b**) TGA of dodecyl amine functionalized GQDs **5** (red) and dodecyl amine **4** (blue) (**c**) ^1^H-NMR spectrum of dodecyl amine functionalized GQD **5** in toluene-d_6_.

In order to obtain the desired material graphene quantum dots (GQDs) were prepared according to a modified literature procedure^39^. Flaked graphite was treated with H_2_SO_4_ and HNO_3_ at 120 °C for 24 h as depicted in Fig. 1a. The resulting material GQD_COOH_ **2** was analyzed by XRD, Raman and AFM (see supporting information). In order to functionalize the carboxylic acid groups at the edges of graphene sheets, the carboxylic acid functions were first transferred into carboxylic acid chloride groups. Therefore, the material was treated with oxalyl chloride as shown in Fig. 1a. The modified material GQD_COCl_ **3** was analyzed (see supporting information). Due to the moisture sensitive carboxylic chloride functions, GQD_COCl_ **3** agglomerated on the AFM waver. Hence GQD_COCl_ **3** was directly used in the subsequent step. The as-prepared material was treated directly with dodecyl amine **4** in order to form the desired carboxylic acid amides (Fig. 1a). With dodecyl amine **4** a high level of functionalization was achieved. The obtained material GQD_Dodecylamide_ **5** was analyzed. After the functionalization of GQD_COOH_ **2** with dodecyl amine a complete solubility in solvents like ethers, methanol, *n*-hexane, heptane, xylene, dichloromethane and toluene was observed. The pure GQD_COOH_ **2** was insoluble in these solvents.

In order to evaluate the behavior under high temperature the resulting solid was analyzed by TGA (Fig. 1b) (for TGA comparing **2**, **3** and **5** see supporting information). The GQD dodecyl amine compound **5** showed a higher thermal resistance than pure dodecyl amine^46^. Due to the high solubility of the material in different solvents (for pictures of solutions with different solvents see supporting information) an ^1^H-NMR spectrum in deuterated toluene showed the formation of an amide group with a signal at 7.9 ppm in deuterated toluene (Fig. 1c) and 8.08 ppm in deuterated methanol (see supporting information). Furthermore, XRD showed a structured orientation of the dodecyl chains (see supporting information). The degree of amide formation was determined by the content of nitrogen and oxygen by elementary analysis (w.%) (C = 71.9%; N = 6.5%; O = 11.3%; H = 11.4%), which corresponds to 80 mol%. An XPS study showed similar atomic ratios (see supporting information).

**Figure 2:**
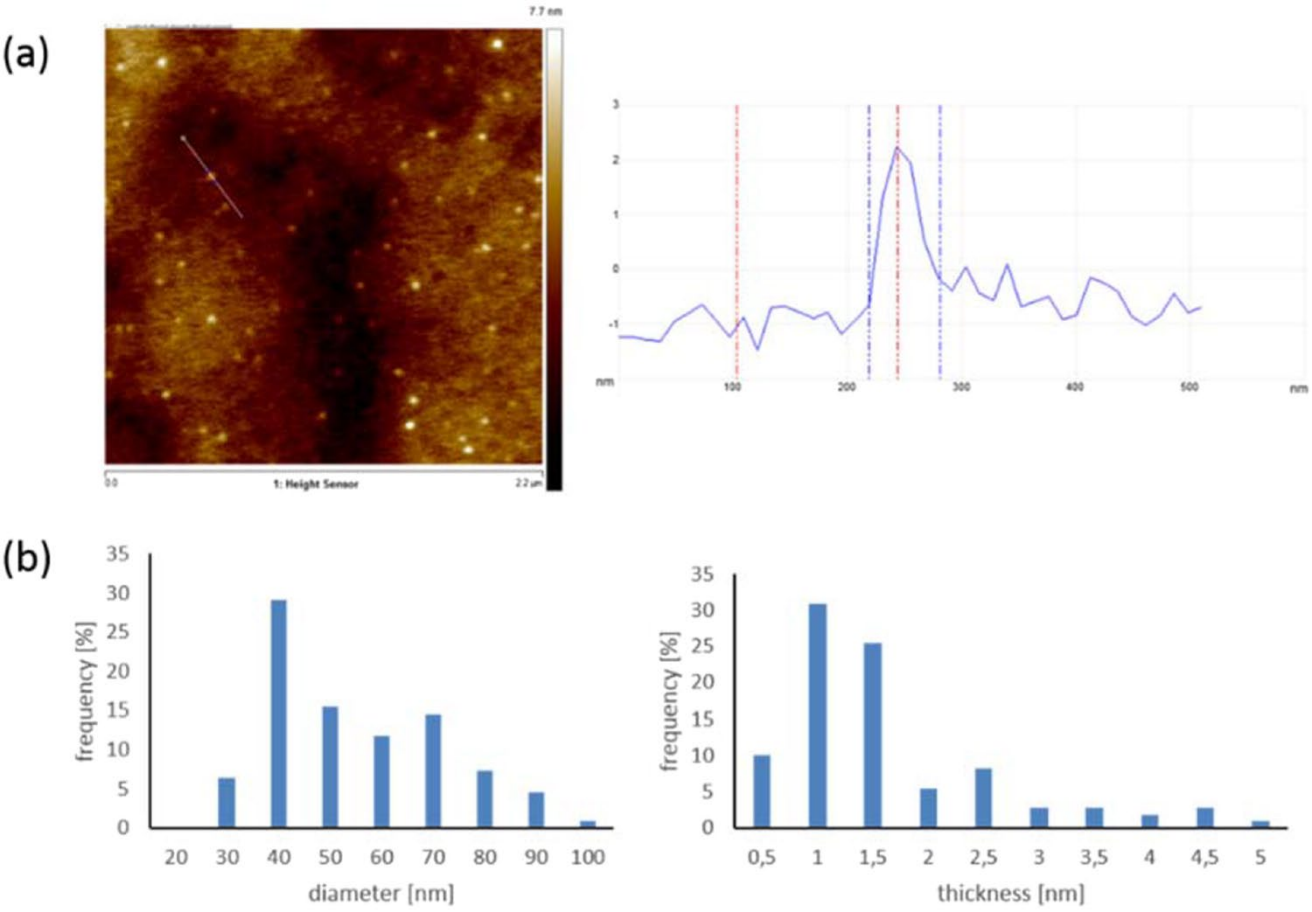
(**a**) AFM height image and height measurement (**b**) diameter and thickness distribution of 110 measured particles.

Atomic force microscopy measurements of **5** revealed that the single particle has an average size of 38 nm and an average thickness of 1.5 nm (Fig. 2). Compared to material **3** an exfoliation was observed. In addition, the average number of layers in sheets was ca. 2 as shown by AFM. Graphene particles with these average lateral dimensions are defined as graphene quantum dots^47,48^.

**Figure 3:**
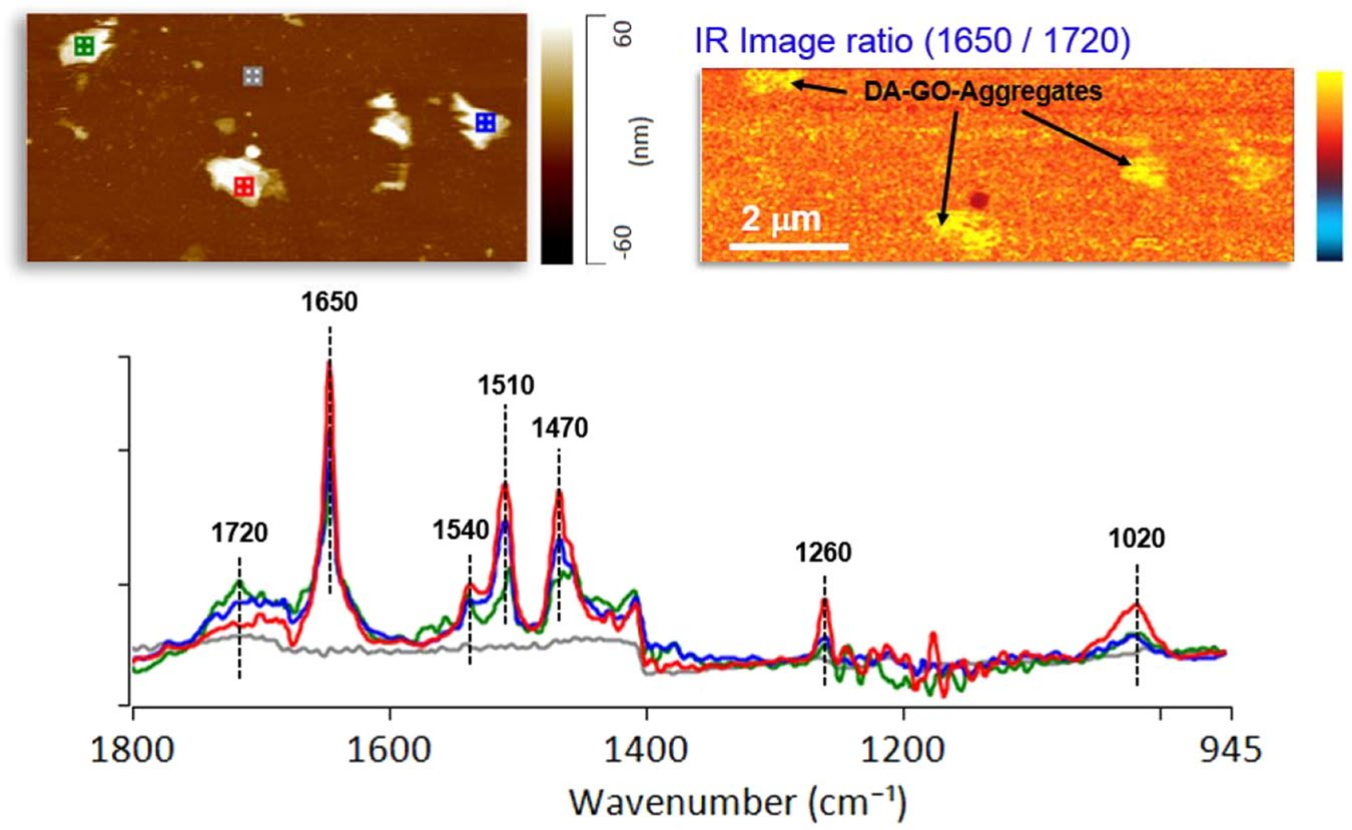
(**a**) AFM height image (**b**) the AFM-IR ratio of 1650/1720 cm^−1^ and (**c**) IR spectrum of the selected areas of (**a**).

For the identification of the sucessful amide formation an additional atomic force microscope infrared-spectroscopy measurment was performed (Fig. 3). It showed that **5** contained the expected amide functions. Figure 3 confirms that the small dots were dodecyl amine functionalized GQDs **5**. The functional groups are covering partialy the GQD surface. When the GQDs form agglomerates they are covered completely by the groups. It was possible to identify functional groups by the IR data, including an epoxide stretch (C-O-C) at 1020 cm^−1 ^^49^, amide I at 1650 cm^−1 ^^49^, amide II at 1540 cm^−1 ^^49^, amide III at 1260 cm^−1^ ν (CH_2_), and a C=O stretch at 1720 cm^−1^. (For a comparative FTIR study of **2**, **3** and **5** see supporting information).

**Figure 4:**
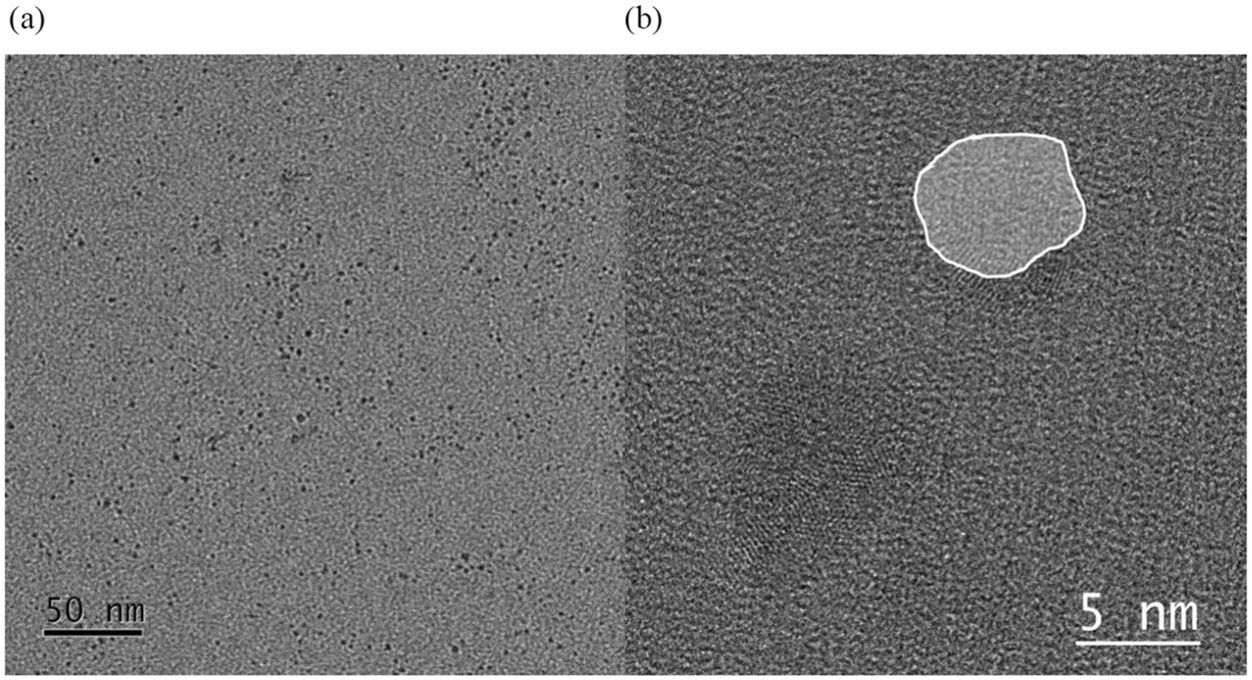
(**a**) TEM bright-field overview of GQDs **5** (**b**) HRTEM of two quantum dots (one highlighted) showing a crystalline lattice.

In addition, GQDs **5** was investigated via HRTEM. An overview of the sample is shown in Fig. 4(a). Now, it is also possible to see smaller particles with a diameter of 5–10 nm compared to the AFM measurement. HRTEM shows the crystalline lattice of the GQDs (Fig. 4(b)).

**Figure 5:**
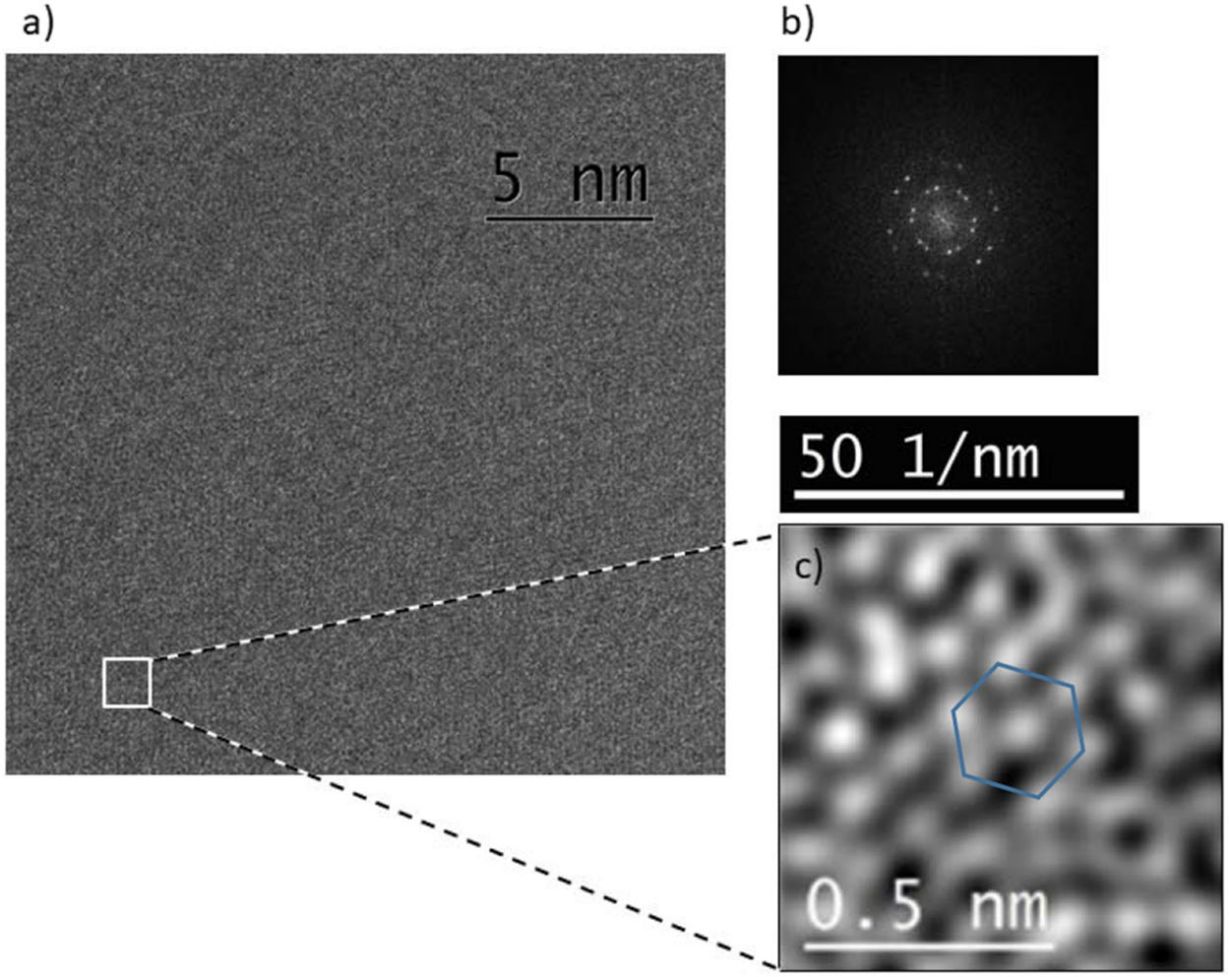
(**a**) HRTEM bright-field overview and fast Fourier transform of GQDs **5**. (**b**) Spot distances of the inner two rings correspond to 0.208 nm and 0.120 nm. (**c**) Fourier filtered close-up of (**a**) showing averaged nearest neighbour distances of 0.137 nm.

This is further supported by a HRTEM structure determination depicted in Fig. 5. The lattice contrast is barely visible in the HRTEM image due to the 11 nm thick carbon support foil. Hence, the crystalline structure of the sample was visualised by a Fourier transformed image (Fig. 5b). The Fourier filtered image in Fig. 5(c) using the intensity of all spots on the inner two rings in Fig. 5(b) shows the regular arrangement of atomic positions. The six-fold symmetry of the lattice is highlighted. From this a mean atomic next neighbor distance of 0.137 nm can be determined, in reasonable agreement with the bond length of 0.142 nm for sp^2^ hybridized carbon in graphene and smaller bond lengths reported for C=C bonds at lower hybridization^50^. The thickness of individual GQDs was determined by energy-filtered TEM using the log-ratio method and electron mean free path estimations according to the Malis model^51^ to be 2–3 nm.

**Figure 6:**
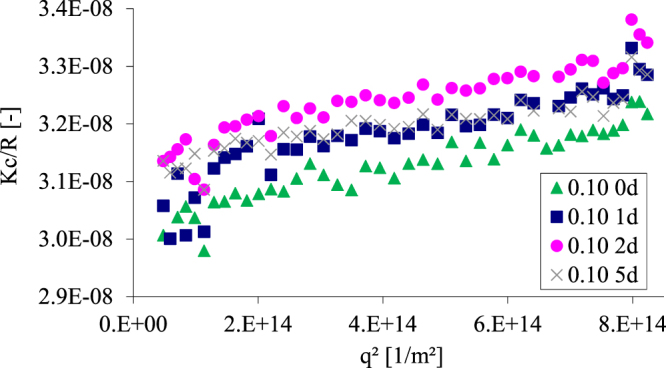
Time dependence of scattering intensities (in a.u.) obtained by static light scattering of a dispersion of 0.1% (mass percent) dodecyl amine modified GQDs **5** for 0 d (green, triangle), 1d (blue, square), 2d (pink, circle) and 5d (gray, cross).

In order to evaluate the solution stability of the dodecyl amine functionalized GQDs **5**, the latter were dissolved in toluene and the resulting solution was observed for several days with static light scattering (Fig. 6). It was found that the particle size remained constant over the measured period. The deviation was within the range of the standard deviation of static light scattering.

**Figure 7:**
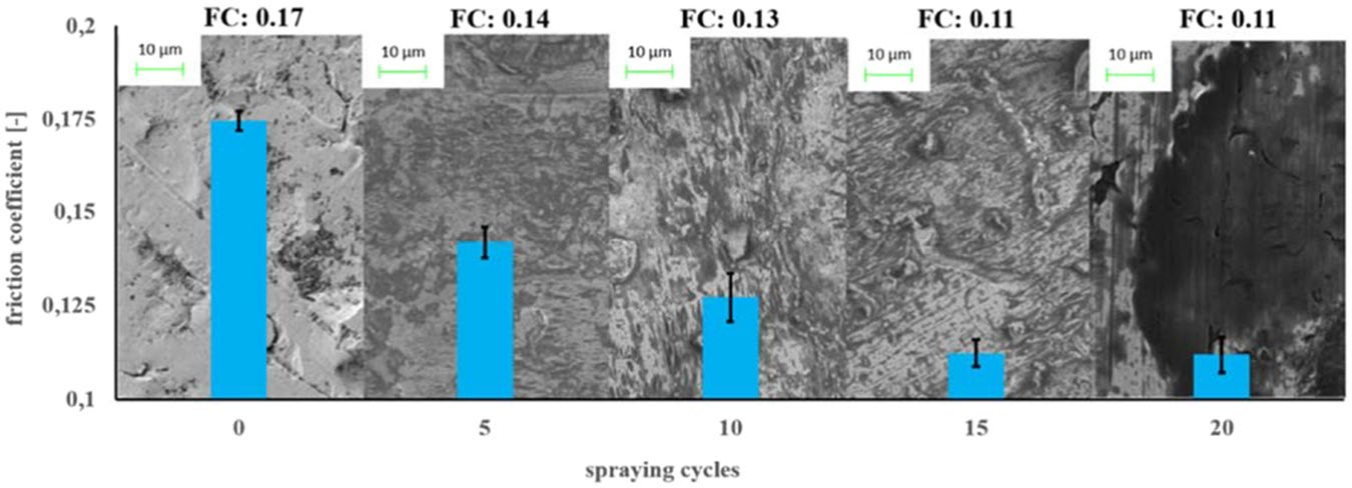
SEM images of steel samples coated by a different number of spray cycles of dodecyl amine functionalized GQDs **5** and corresponding friction measurements.

In order to explore the potential of **5** as lubricant, the resulting dodecyl amine GQD **5** solution was sprayed on a fresh cleaned steel surface and the lubrication was measured with different surface coverage (Fig. 7). The surface coverage was controlled by the number of spray cycles, with the result, that the friction coefficient is decreasing with an increasing number of spray cycles. The macroscopic friction behavior was investigated with a Thwing-Albert FP-2250 friction tester. The pure and rough steel surface had a friction coefficient of 0.17. With an increasing number of spray cycles up to 20 the friction coefficient decreased to a value of 0.11.

**Figure 8:**
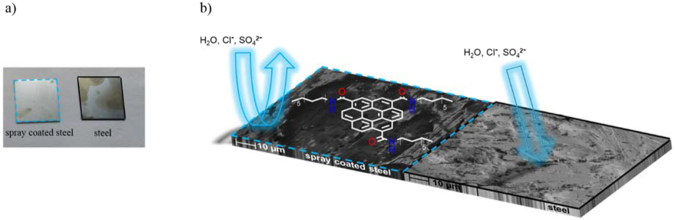
(**a**) Image of steel samples after immersing 1 h in sea water. Right: untreated sample, left: steel with 20 spray cycles of dodecyl amine functionalized GQDs **5**. (**b**) Schematic mechanism of the corrosion inhibition with SEM images of steel with 20 spray cycles of dodecyl amine functionalized GQDs **5** and untreated steel.

With an increased number of spraying cycles, the steel surface is more and more covered, which is a requirement for corrosion protection. The corrosion behavior was investigated by a steel sample with 20 spray cycles, which was emerged for 1 h in sea water (based on DIN 51358). The result is depicted in Fig. 8. The sample with 20 spray cycles showed only a small attack by corrosion. **5** inhibited the corrosion but could not protect against corrosion completely.

**Figure 9:**
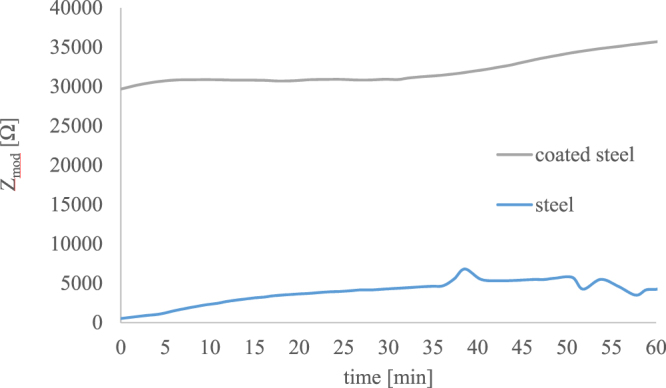
Time dependent absolute values of the impedance at a frequency of 0.1 Hz as a function of the exposure time in artificial seawater for the coated steel and the bare steel.

Based on the time-dependent measurements of the impedance at 0.1 Hz (Fig. 9), it gets obvious that the corrosion resistance of the GQDs **5** coated steel is about an order of magnitude higher than that of uncoated steel. In both case the corrosion resistance slightly increases over time most probably based on the formation of corrosion products that slow down the interfacial transfer reactions. The impedance measurements thereby support the qualitative observations of the seawater immersion test^52^.

Hence, **5** applied as a lubricant can inhibit tribo-corrosion. The protecting mechanism is resulting from a barrier property of the dodecyl amine functionalized GQDs **5** film. Taking into consideration that the film thickness is at 20 spray cycles only 17 nm (as determined by ellipsometry measurements, see supporting information) the resulting effect is remarkable. Often a higher coating thickness is found with other carbon based coatings based on graphite^10^.

## Conclusion

Graphene quantum dots (GQDs) and dodecyl amine functionalized GQDs have been prepared and investigated as lubricant. The resulting modified GQDs showed very good solubilities in numerous organic solvents, including ethers, methanol, n-hexane, heptane, xylene, dichloromethane and toluene. The good solubility provided the possibility of an efficient and fast spray application of the functionalized GQDs to a steel surface. In the presented work, it was possible to show with different methods that the successful covalent modification of GQDs with dodecyl amine was achieved. The resulting particles had an average size between 5 and 40 nm. The functionalized GQDs could be applied in spray coating to create a thin lubricant film on steel. The resulting lubricant film reduced the friction coefficient from 0.17 to 0.11 on the macro scale and it was shown for the first time that this graphene quantum dot based material has a significant corrosion inhibition effect. Compared to unfunctionalized graphene oxide, the functionalisation caused a significant enhancement of the lubricant^10^. In addition, a better friction coefficient was observed compared to alkylated graphene oxide systems in combination with oil^11^. The presented results will help to further improve the development of tribo-corrosion inhibiting lubricant systems based on graphene derivatives.

## Electronic supplementary material

Supplementary Information
