# Supplementary material for: A Novel Lubricant Based on Covalent Functionalized Graphene Oxide Quantum Dots
Source: Sci Rep. 2018 Apr 11;8:5843. doi: 10.1038/s41598-018-24062-2 (PMC5895846; doi:10.1038/s41598-018-24062-2)
Supplement: Supplementary file 1 — Supplementary Information [file 41598_2018_24062_MOESM1_ESM.pdf]

Supporting Information

A Novel Lubricant Based on Covalent Functionalized Graphene Oxide Quantum Dots

Andreas Wolk,[1] Marta Rosenthal,[2] Stephan Neuhaus,[1] Klaus Huber,[3] Katharina Brassat,[4] Jörg K. N. Lindner,[4] Richard Grothe,[5] Guido Grundmeier,[5] Wolfgang Bremser,[1] & René Wilhelm[2]

[1]University of Paderborn, Department of Chemistry, Technical Chemistry, Warburgerstr. 100, 33098 Paderborn, Germany. [2]University of Paderborn, Department of Chemistry, Organic Chemistry, Warburgerstr. 100, 33098 Paderborn, Germany. [3]University of Paderborn, Department of Chemistry, Physical Chemistry, Warburgerstr. 100, 33098 Paderborn, Germany. [4]University of Paderborn, Department of Physics, Warburgerstr. 100, 33098 Paderborn, Germany. [5]University of Paderborn, Department of Chemistry, Technical Chemistry, Warburgerstr. 100, 33098 Paderborn, Germany. Correspondence and requests for materials should be addressed to R.W. (email: rene.wilhelm@uni-paderborn.de) or W.B. (email: wolfgang.bremser@uni-paderborn.de)

**AFM of GQD_{COOH} 2**	**S2**
**RAMAN of GQD_{COOH} 2**	**S3**
**AFM of GQD_{COCl} 3**	**S4**
**^{1}H-NMR of $GQD_{Dodecylamide}$ 5**	**S5**
**Comparative FTIR of 2, 3 and 5**	**S6**
**Comparative TGA of 2, 3 and 5**	**S8**
**Pictures of 2, 3 and 5 in different solvents**	**S9**
**Ellipsometry study after 20 spray cycles**	**S10**
**XPS Study of $GQD_{Dodecylamide}$ 5**	**S11**
**XRD of $GQD_{Dodecylamide}$ 5**	**S12**
**XRD of $GQD_{Dodecylamide}$ 5, GQD_{COOH} 2 and pure dodecylamine 4**	**S13**
**Energy-filtered TEM thickness**	**S14**

AFM of GQD_{COOH} 2

The material was analysed by AFM and contained sheets with an average diameter of 60 nm as shown in Figure S1. The average height of the sheets was 1.211 nm, which would indicate that few-layer graphene quantum dot with three layers were obtained. The latter are abbreviated as GQD_{COOH} **2**.

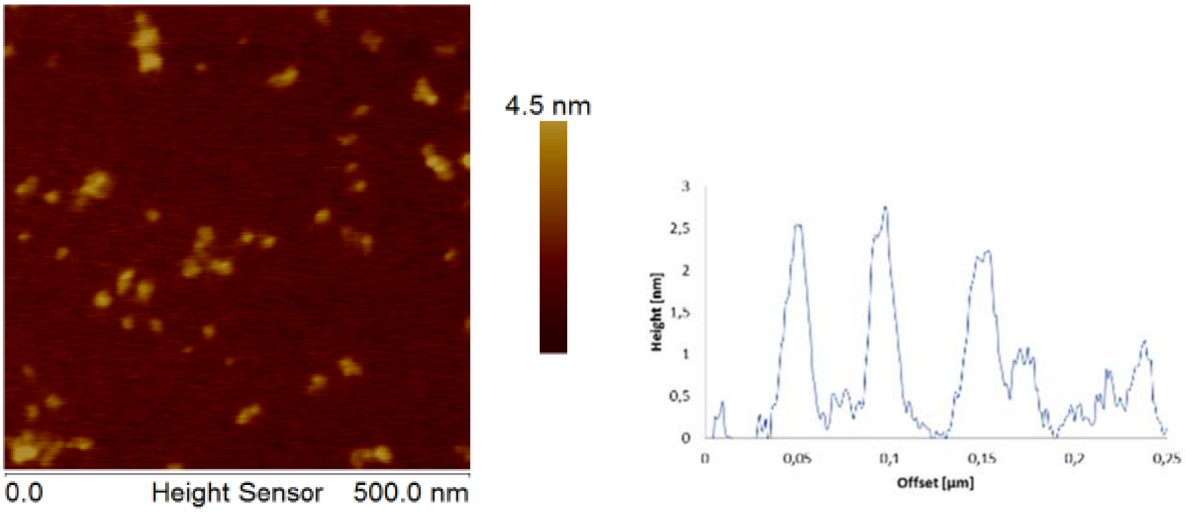

Pair	Horizontal Distance [µm]	Vertical Distance [nm]
**1**	0.048	1.871
**2**	0.064	1.730
**3**	0.032	1.382
**4**	0.080	1.441
**5**	0.080	0.230
**6**	0.032	0.312
**7**	0.080	0.156
**8**	0.032	0.780
**9**	0.064	2.180
**10**	0.048	2.028
∑	0.059	1.211

Figure S1. AFM images of GQD_{COOH} **2** measured on mica surface.

RAMAN of GQD_{COOH} 2

A Raman spectrum revealed the formation of the desired graphene material as shown in Figure S2. As can be seen from the Raman, a few defects were present on the surface of the sheets. Yet, the presence of the 2D signal and a relative small D signal showed that only few defects were present on the surface of the graphene quantum dots compared to graphene oxide prepared after the Hummers method, where large defects result in a very strong D peak and the vanishing of the 2D peak.[1]

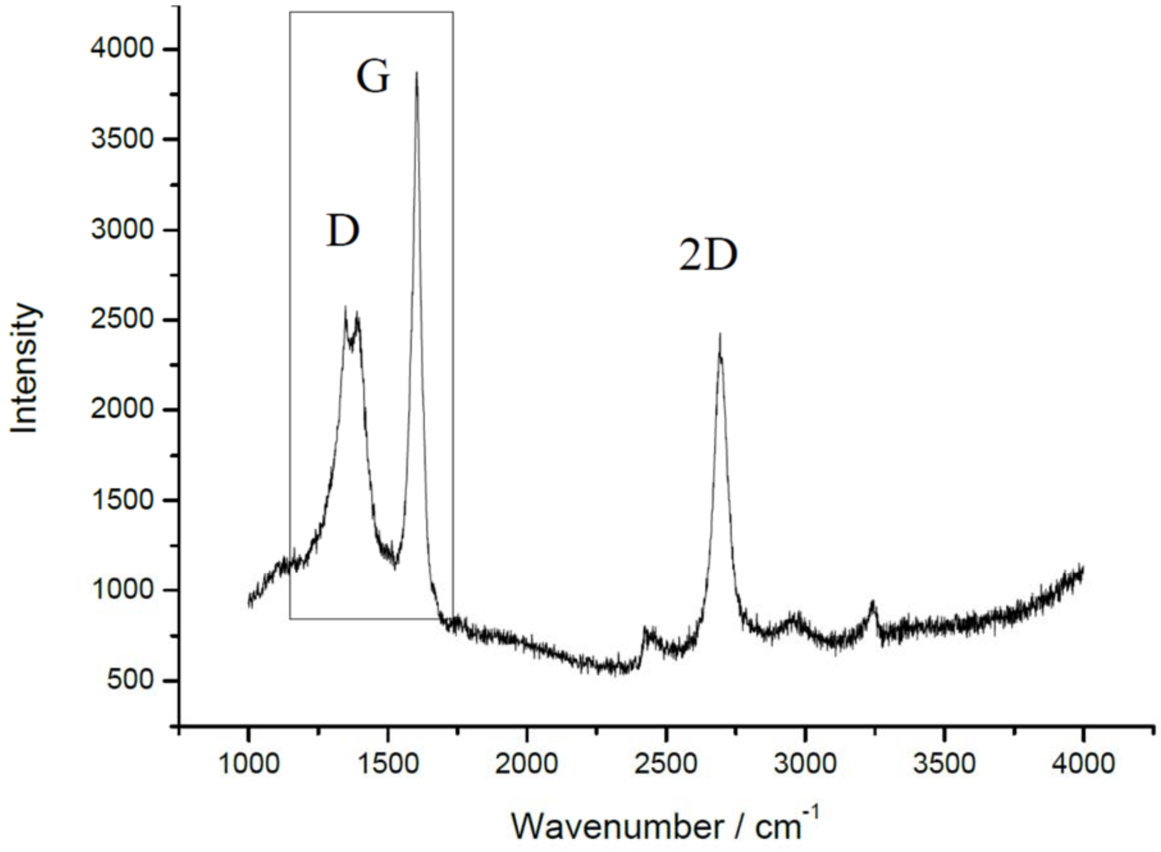

Figure S2. Raman of GQD_{COOH} **2**

AFM of GQD_{COCl} 3

GQD_{COCl} **3** was analysed by AFM as depicted in Figure S3. During the treatment, the size of the sheets increased and also the number of layers increased to ca. 9. This could be also attributed to the sample preparation for microscopy. In the presence of small amounts of water carboxylic acid chlorides hydrolyse to the carboxylic acid functions, which could react with the remaining carboxylic acid chloride functions to anhydrides. If different sheets are reacting a larger agglomerate could be formed.

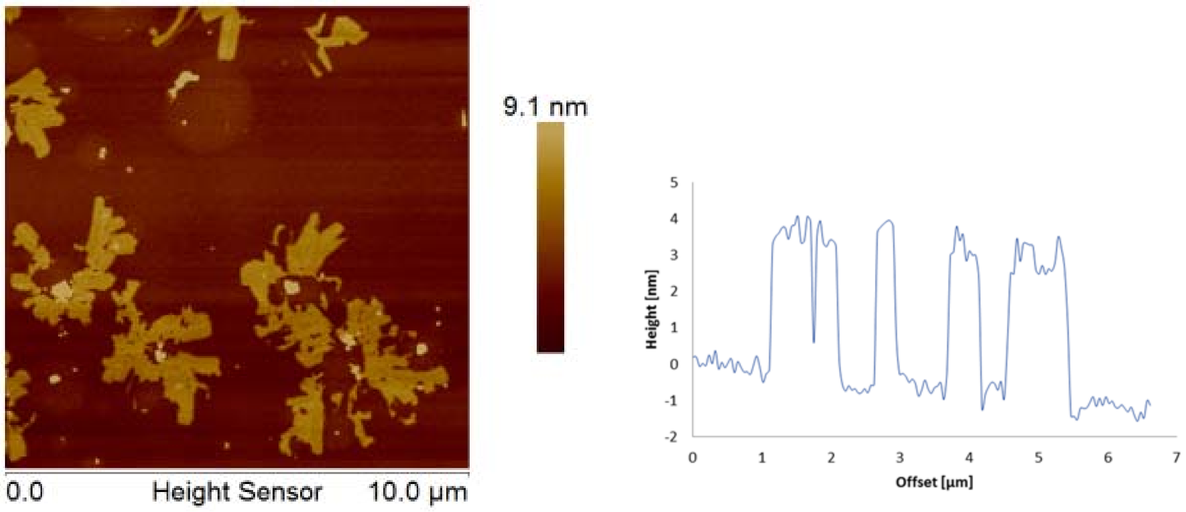

Pair	Horizontal Distance [µm]	Vertical Distance [nm]
**1**	1.096	4.438
**2**	0.817	3.593
**3**	2.170	3.703
**4**	1.553	3.841
**5**	1.872	3.703
**6**	1.894	4.264
**7**	2.233	4.567
**8**	1.261	3.722
**9**	1.056	3.915
**10**	1.343	4.673
**Σ**	1.529	4.042

Figure S3. AFM images of GQD_{COCl} **3** measured on mica surface.

^{1}H-NMR of GQD$_{\text{Dodecylamide}}$ 5

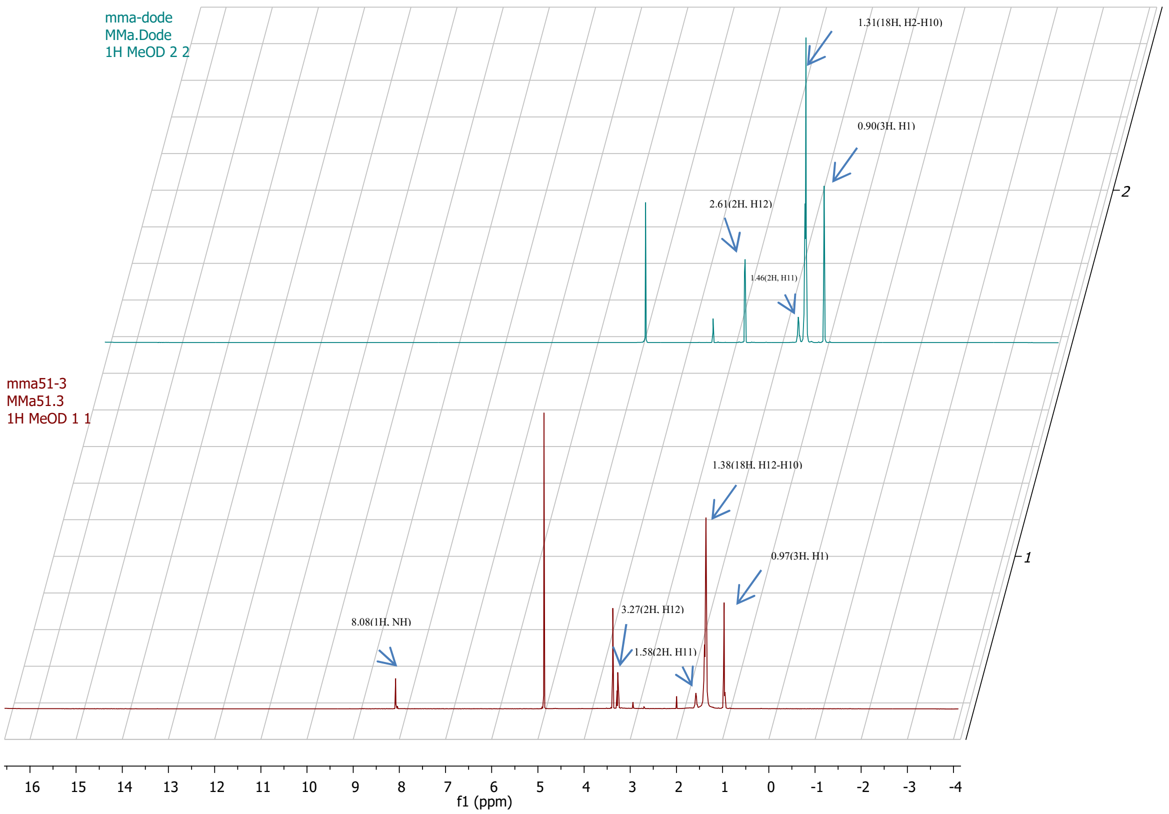

Figure S4. ^{1}H-NMR of GQDDodecylamide **5** in deuterated MeOH (below) and for comparison dodecyl amine **4** (above).

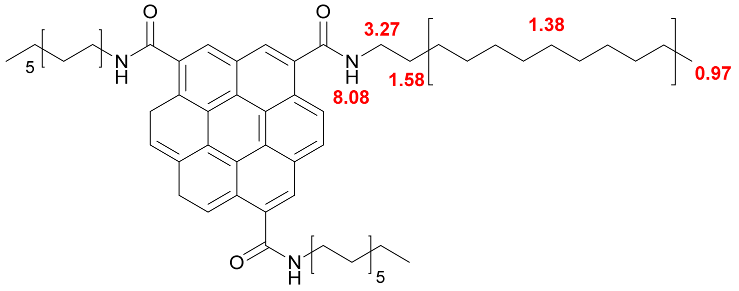

GQD$_{\text{Dodecylamid}}$ **5**

^{1}H-NMR (500 MHz, CD_3OD-d_6): δ [ppm] = 0.97 (t, J = 7.0 Hz, 3 H, H-1), 1.38 (s, 18 H, H-2, H-3, H-4, H-5, H-6, H-7, H-8, H-9, H-10), 1.53-1.63 (m, 2 H, H-11), 3.27 (t, J = 7.0 Hz, 2 H, H-12), 8.08 (s, 1 H, NH).

IR (KBr) $\boldsymbol{\nu}$ = 665, 777, 1251, 1350, 1498, 1644, 1734, 2953, 3290 cm^{-1}.

Comparative FTIR of 2, 3 and 5

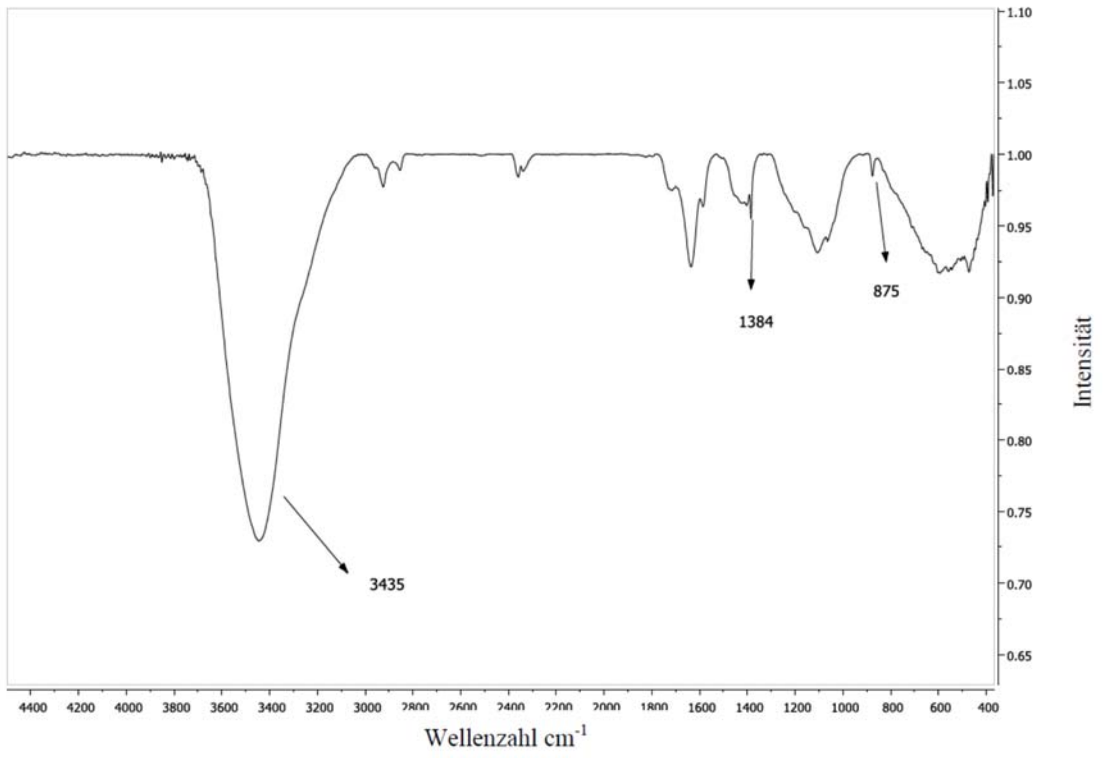

Figure S5. IR of GQD_{COOH} **2** in KBr

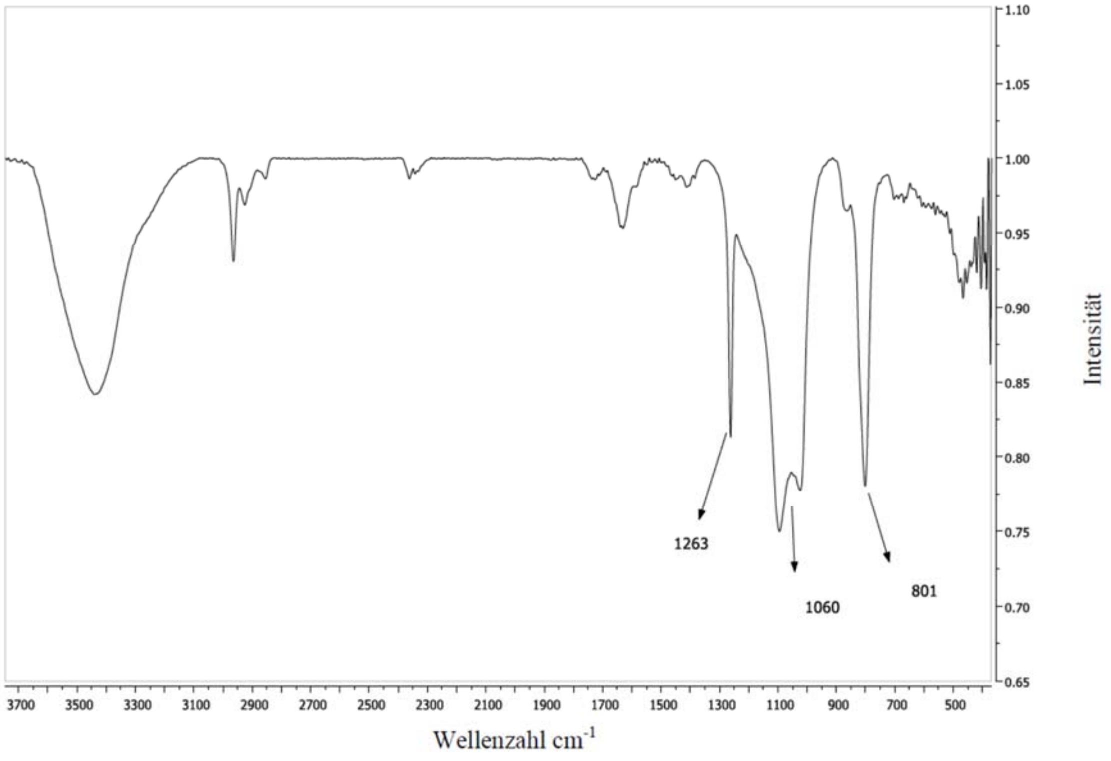

Figure S6. IR of GQD_{COCl} **3** in KBr

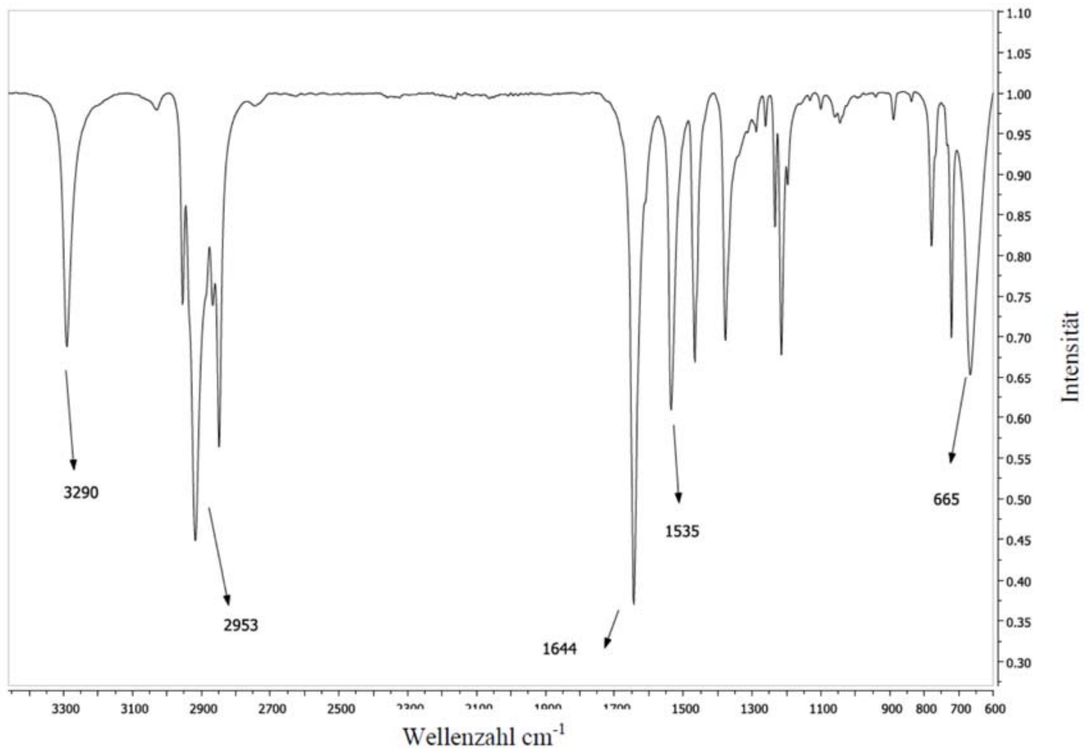

Figure S7.IR of GQD$_{Dodecylamide}$ **5** in KBr

Comparative TGA of 2, 3 and 5

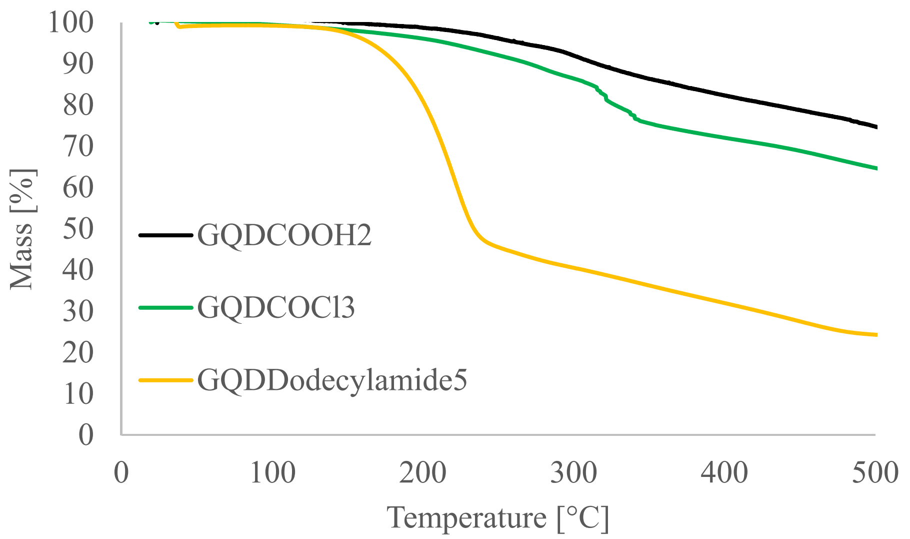

Figure S8. TGA of GQD_{COOH} **2**, GQD_{COCl} **3** and $GQD_{Dodecylamide}$ **5**

Pictures of 2, 3 and 5 in different solvents

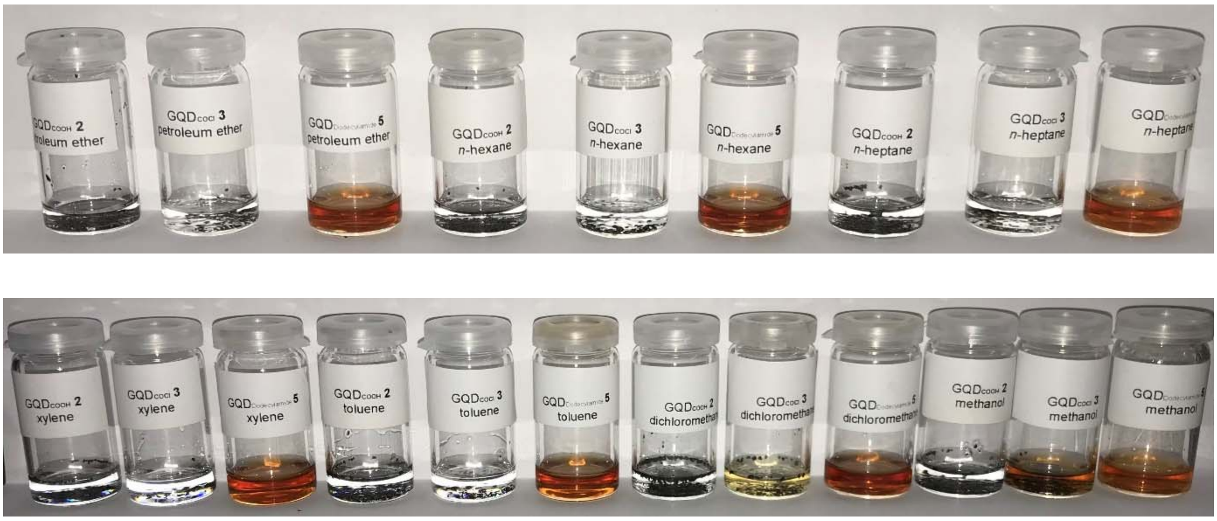

Figure S9. **2**, **3** and **5** in different solvent after 1 h.

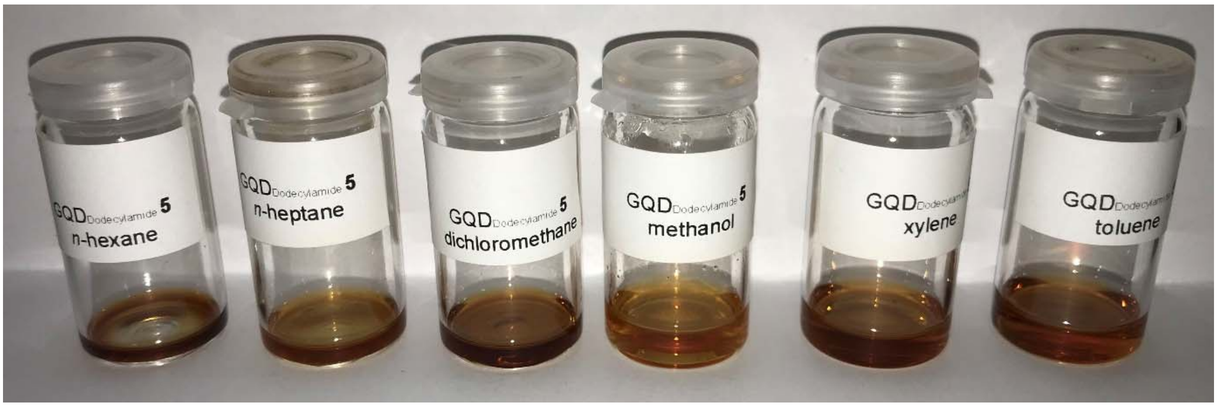

Figure S10. GQD$_{\text{Dodecylamide}}$ **5** in different solvents after 28 days

Ellipsometry study after 20 spray cycles

- Exsitu Accurion Imaging Ellipsometer nanofilm_ep4 (Software: EP4).

- Two variations: AOI_ and Lambda_variations.

- For graphene: two models used: Cauchy and Amorphous Carbon models.

Cauchy model (B_n = 3000; C_n = 0)

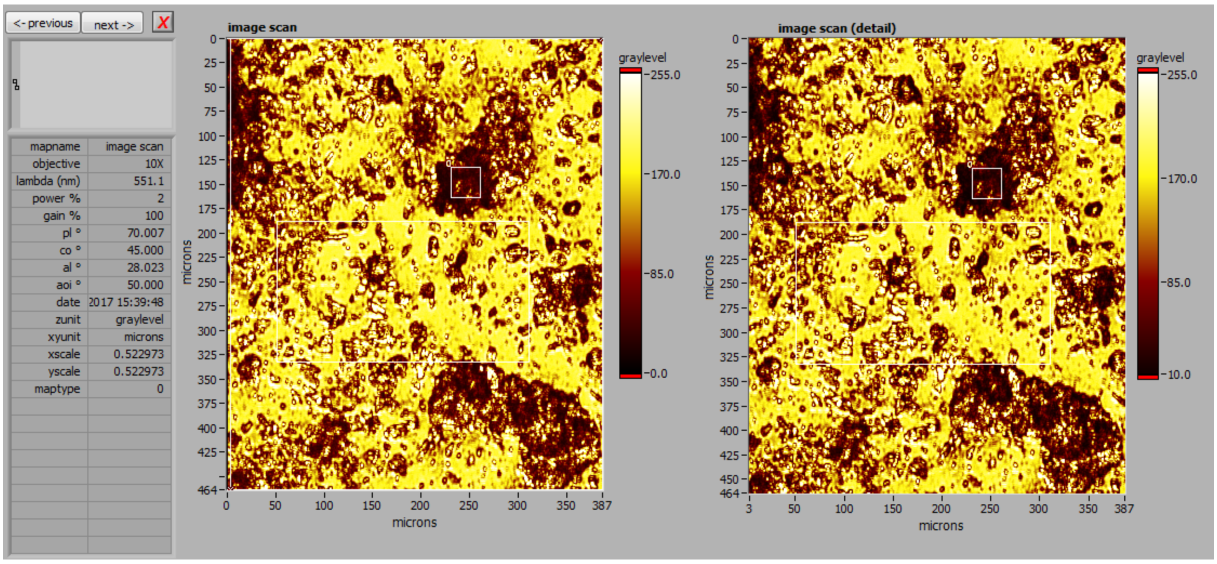

- AOI_Variation (λ=658 nm, AOI=45-70°, AOI step=1°)

Cauchy model			
**Position**	**graphene thickness [nm]**	**graphene A_n**	**RMSE**
1	17.7	2.628	1.593
2	17.0	2.789	2.014

XPS Study of GQD$_{Dodecylamide}$ 5

	C / at%	O / at%	N / at%	Si / at%
GQD5	72.2	13.6	4.2	10.0

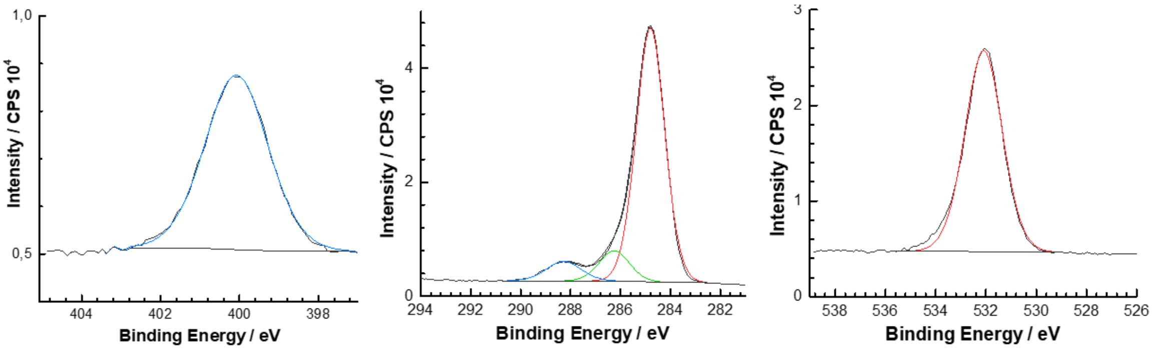

2

Figure S11. XPS high-resolution N1s, C1s and O1s spectra of **5**

XRD of GQD$_{\text{Dodecylamide}}$ 5

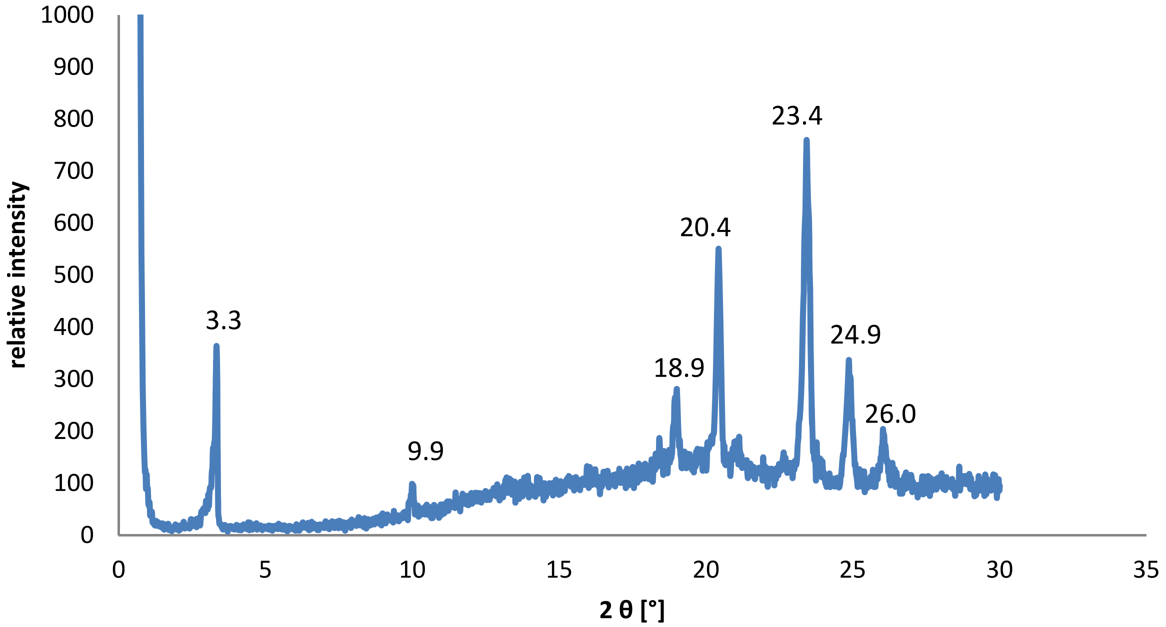

Figure S12. XRD of **5**. Peaks originate from the dodecyl chains

XRD of GQD$_{Dodecylamide}$ 5, GQD$_{COOH}$ 2 and pure dodecylamine 4

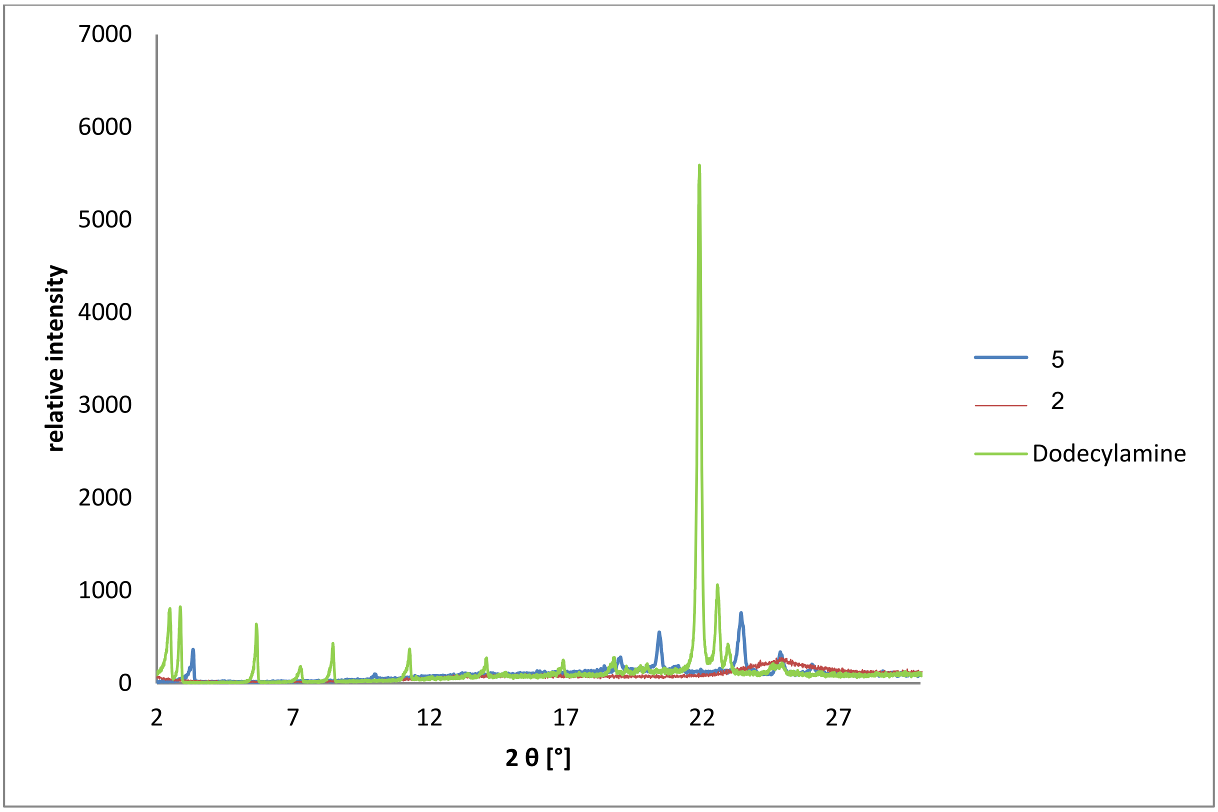

Figure S13. XRD of GQD$_{Dodecylamide}$ **5**, GQD$_{COOH}$ **2** and pure dodecylamine **4**

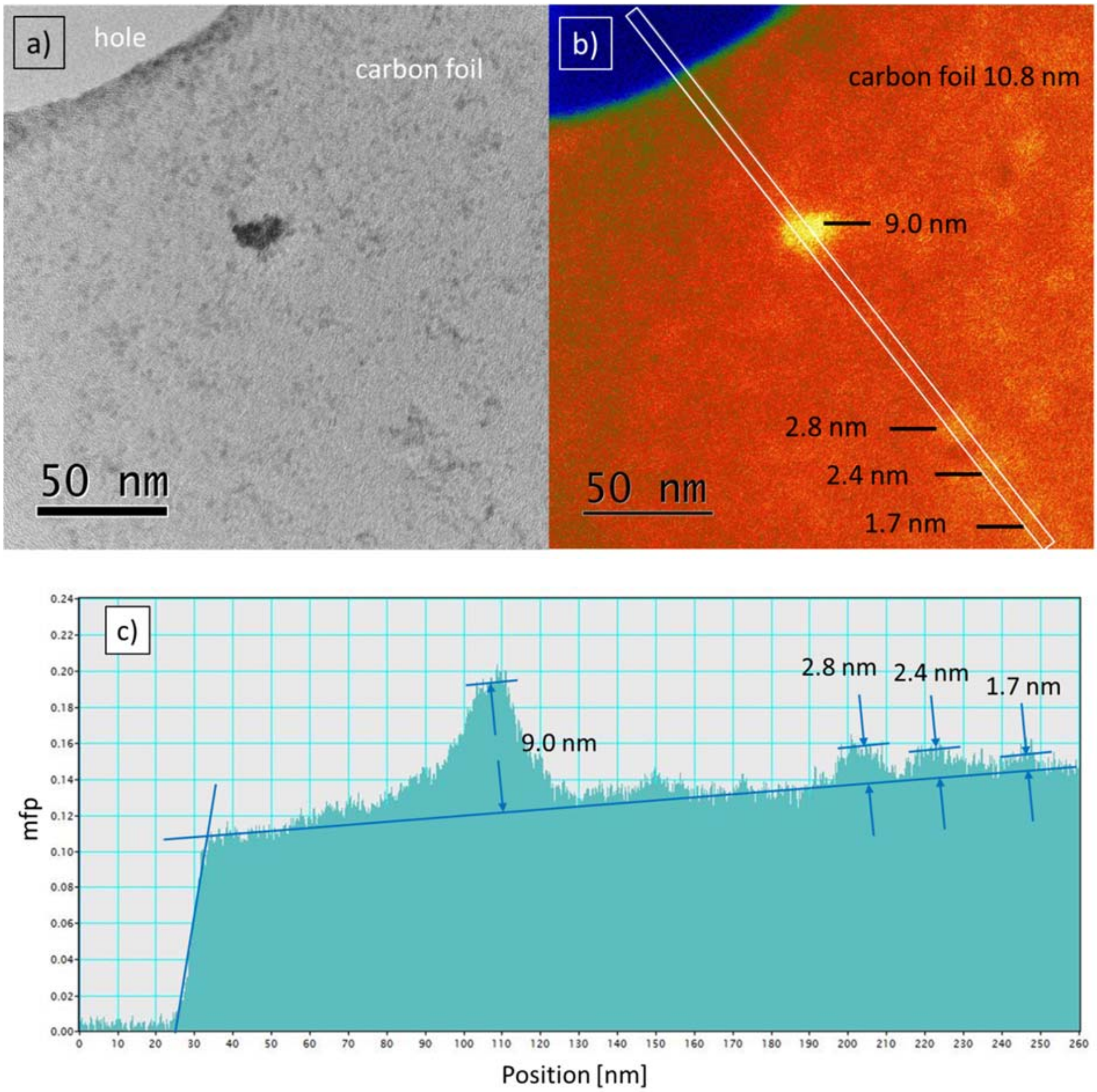

Figure S14

a) TEM bright-field image of graphene oxide quantum dots GQD 5 on a holy carbon foil. b) Energy-filtered TEM thickness map of the same area, calculated by the log-ratio method. Using the Malis model [2] for the estimation of the mean free path (mfp) of 200 keV electrons, the foil thickness and the thickness of quantum dots is calculated. Using the GQD composition determined by elemental analysis, the thickness of GQDs and GQD agglomerates can be determined as well. C) Thickness line profile (in terms of mfp's) along the white line in (b), integrated over the width of the line. From this, the GQD thicknesses were estimated as indicated in (b).

References

1 Eigler, S. Controlled Chemistry Approach to the Oxo-Functionalization of Graphene. *Chem. Eur. J.* **22**, 7012-7027 (2016).

2 Malis, T., Cheng, S. C. & Egerton, R. F. EELS Log-Ratio Technique for Specimen-Thickness Measurement in the TEM. *J. Electron Microsc. Tech.* **8**, 193-200 (1988).
